# Comparative Analysis of Structural Characterisation and Gel Properties of Blended/Co-Precipitated Soy-Pea Dual-Protein

**DOI:** 10.3390/foods14162867

**Published:** 2025-08-19

**Authors:** Lu Wang, Xinyu Zhang, Xinhui Wang, Aiting Hui, Fengying Xie, Xia Wu

**Affiliations:** College of Food Science, Northeast Agricultural University, Harbin 150030, China; 17860540154@163.com (L.W.); zhangxinyu297@163.com (X.Z.); wxh3225@163.com (X.W.); 18846145886@163.com (A.H.)

**Keywords:** soy protein isolate, pea protein isolate, co-precipitated protein, protein–protein interaction, gel properties

## Abstract

This study proposed a pH-driven co-precipitation strategy to overcome the limitations of traditional physical blending in functional improvement of a dual-protein system. The results demonstrated that, in comparison with the soy-pea blended protein (SPBP), the soy-pea co-precipitated protein (SPCP) showed a decrease in α-helix and β-sheet content, accompanied by in an increase in random coil structure. SPCP exhibited decreased fluorescence intensity, smaller particle size (from 392.2 to 176.1 nm) with increased absolute zeta-potential values (from −13.7 to −19.7 mV), reduced surface hydrophobicity (from 21,987.3 to 9744.8), and increased content of disulfide bonds. Structural optimization of SPCP significantly bolstered intermolecular interactions between SPI and PPI. Molecular docking simulations also validated the presence of abundant hydrophobic interactions and hydrogen bonds within in the blend system. These modifications significantly enhanced the solubility of SPCP (especially SPCP8.0). The rheological analysis further revealed that the storage modulus (G′) and loss modulus (G″) of SPCP8.0 were both higher than those of SPBP, while its tan δ was lower than that of SPBP, indicating synergistic interactions between proteins. These interactions contributed to the formation of a more stable three-dimensional network structure, thereby conferring it with superior gel properties. These findings provide theoretical foundations for improving the functional properties of plant-based dual-protein and their applications in plant-based meat production.

## 1. Introduction

Dual-protein food systems involve integrating proteins from different sources, taking advantage of their combined structural and functional properties to overcome the limitations of using single proteins to enhance the food’s functional features (e.g., gelation). Soy protein isolate (SPI) is a kind of plant-based protein, primarily composed of β-conglycinin (7S) and glycinin (11S) globulins. SPI exhibits insufficient molecular flexibility and heterogeneous network formation during gelation, leading to coarse gel texture, which restricts its utilization in food processing [1]. Vicilin (7S) and legumin (11S) are the major components in pea protein isolate (PPI). The relatively loose molecular structure and fewer disulfide bonds in PPI enable it to be added to plant-based meat to improve the texture and elasticity of the gel [2]. Thus, combining these two proteins represents an effective and feasible approach to compensate for the deficiencies in structural and gel properties of a single protein.

Co-precipitated proteins have been prepared through isoelectric precipitation, which modifies the structure of polypeptide chains to form co-assembled proteins with higher order structures, thereby bolstering the proteins’ functional characteristics (e.g., water-holding capacity, gel properties, and nutrition) [3,4]. Among them, polypeptide chains fully unfold when the pH of the mixtures shifts toward basicity, and then gradually refold when the pH is adjusted back to neutral conditions [5]. This structural reorganization process creates favorable conditions for interactions between heterologous proteins, potentially enhancing the solubility and gelation characteristics of the co-precipitated proteins. For instance, Tian, et al. [6] found that soybean-wheat co-precipitated proteins can form denser, more elastic gel networks through hydrogen bonds, hydrophobic interactions, and disulfide bonds. Zhou, et al. [7] confirmed that co-precipitated proteins formed from pea protein and grass carp proteins through disulfide bonds, electrostatic interactions, and hydrophobic forces effectively enhanced solubility and gelation properties of the proteins. These studies primarily focused on functional enhancements but lacked systematic comparisons between physical blending and co-precipitation mechanisms. Particularly, legume-legume co-precipitation remained virtually unexplored, with no reports on SPCP’s structural and gel properties, nor sufficient investigation into their industrial application mechanisms in plant-based meat products. Li, et al. [8] also showed that the pH adjustment during soy-walnut protein co-assembly promoted structural unfolding/refolding, and facilitated the creation of a more ordered and denser gel network through hydrogen bonds and hydrophobic interactions. In addition, Choi, et al. [9] discovered that the incorporation of PPI weakened the rigid structure of SPI, which in turn optimized their gel properties to a certain extent. However, simple physical blending was often insufficient to achieve both optimal structural overlap and functional properties between two proteins. Furthermore, Zhou, et al. [7] have demonstrated that proteins extracted under alkaline pH conditions exhibited superior nutritional properties and heat-induced gel properties. However, the optimal effect of specific alkaline pH values on SPCP remains unclear.

Thus, this study aimed to emphasize the crucial role of pH regulation in protein conformational reorganization and its effect on enhancing interactions, and to determine the optimal pH for achieving superior functional properties. For this purpose, SDS-PAGE, fourier transform infrared spectroscopy (FTIR), and intrinsic fluorescence spectrometry were comprehensively employed to analyze the composition and structural characteristics of SPBP and SPCP. Meanwhile, measurements were taken for particle size, zeta potential, surface hydrophobicity, and the contents of sulfhydryl and disulfide bonds, combined with intermolecular interaction studies and molecular docking analysis to thoroughly investigate the protein–protein interaction mechanisms. Furthermore, the solubility and rheological behavior of the coprecipitated protein system were also evaluated. This study provides a theoretical basis for the extensive application of SPCP in the food industry (e.g., plant-based meat).

## 2. Materials and Methods

### 2.1. Materials

Defatted soy flakes were acquired from Shandong Scents Grains & Oil Co., Ltd., located in Binzhou, China. Defatted pea powder was obtained from Wenji Food Co., Ltd. located in Zhaoyuan, China. The remaining chemicals employed were of analytical grade.

### 2.2. Preparation of Soy-Pea Blended Protein (SPBP) and Soy-Pea Co-Precipitated Protein (SPCP)

The acquisition of SPI followed the approach of Wang, et al. [10], with a pH of 7.2 for dissolution in an alkaline solution and a pH of 4.5 for acidic precipitation. According to the method detailed by Zhao, et al. [11], PPI was extracted with pH of 9.0 for alkaline dissolution and 4.5 for acidic precipitation. SPI and PPI were mixed at a mass ratio of 1:1 (*w*/*w*) to obtain the SPBP sample. For the preparation of co-precipitated protein, defatted soy and pea powders (1:1 *w*/*w*) were dissolved in deionized water at a ratio of 1:10 (*w*/*v*). The pH of the solution was then adjusted to 7.2, 8.0, and 9.0 with 2 mol/L NaOH solution, respectively. The mixture was stirred for 2 h at 45 °C before being centrifuged at 8500 rpm for 10 min. The pH of the obtained supernatant was modified to 4.5 with 2 mol/L HCl solution, and the solution was centrifuged again at 8500 rpm for 15 min. Three rinses with distilled water were performed on the collected precipitate. The rinsed precipitate was dissolved and brought to a pH of 7.0 using 2 mol/L NaOH solution, then freeze-dried to obtain SPCP7.2, SPCP8.0, and SPCP9.0 samples.

### 2.3. Sodium Dodecyl Sulphate-Polyacrylamide Gel Electrophoresis (SDS-PAGE)

SDS-PAGE was executed based on the method described by Ma, et al. [12]. 5.0 mg/mL solution of SPI, PPI, SPBP, and SPCP was centrifuged at 9600 rpm for 14 min. The supernatant was blended with 4×Laemmli loading buffer, both containing and lacking DTT, and then subjected to 6 min of heating in a boiling water bath. The gel system consisted of a 5% stacking gel and a 10% separating gel, electrophoresed at 80 V and 120 V, respectively. Finally, the gel imager (Gel Doc EZ, Bio-Rad Laboratories Co., Ltd., Hercules, CA, USA) was used to observe protein bands in gel.

### 2.4. Fourier Transform Infrared (FTIR) Spectroscopy

The samples were analyzed using FTIR spectroscopy according to the method of Dong and Cui [13]. The SPI, PPI, SPBP and SPCP samples were each combined with potassium bromide (KBr) at 1:100 ratio and formed into a disk, respectively. FTIR spectra of the samples were acquired in the range of 400 to 4000 cm^−1^ using an FTIR spectrometer (Nicolet is50, Thermo Fisher Scientific Inc., Waltham, MA, USA). The resolution for measurements was set at 4 cm^−1^, with 32 scans averaged each sample. The amide I band (1600–1700 cm^−1^) in the FTIR spectra was deconvoluted and curve-fitted using PeakFit software (version 4.12, Seasolve Software Inc., San Jose, CA, USA) for secondary structure analysis.

### 2.5. Intrinsic Fluorescence Spectrometry

The initial preparation of SPI, PPI, SPBP, and SPCP samples was at 1.0 mg/mL, followed by dilution to 0.1 mg/mL. Emission wavelengths from 300 to 450 nm were acquired using the fluorescence spectrophotometer (RF-6000, Hitachi Co., Ltd., Tokyo, Japan), with the excitation wavelength set at 290 nm. A 5 nm slit width was applied, and the reaction took 0.2 s.

### 2.6. Surface Hydrophobicity (H_0_)

The determination of H_0_ was carried out using the method described by Wang, et al. [14]. Protein solutions of SPI, PPI, SPBP, and SPCP were prepared at 1.0 mg/mL and then diluted to various concentrations ranging from 0.1 to 0.5 mg/mL. A 4 mL diluted sample solution was combined with 40 μL of ANS at 8.0 mmol/L and incubated in the dark at 25 °C for 15 min. The fluorescence spectrophotometer (RF-6000, Hitachi Co., Ltd., Tokyo, Japan) was employed to assess fluorescence intensity at 390 nm for excitation and 470 nm for emission.

### 2.7. Particle Size and Zeta-Potential

Samples of SPI, PPI, SPBP, and SPCP at concentration of 1.0 mg/mL were analyzed. The granularity analyzer (Zetasizer Nano ZS90, Malvern Instruments Ltd., Malvern, UK) was used to measure particle size and zeta-potential with these parameters: sample refractive index 1.450, solvent refractive index 1.330. The samples were maintained at 25 °C for 2 min before performing triplicate measurements.

### 2.8. Free Sulfhydryl (-SH) and Disulfide Bond (S-S) Contents

The contents of -SH and S-S were determined using the method outlined by Kang, et al. [15], with some adjustments. Briefly, 40 mg samples of SPI, PPI, SPBP, and SPCP were separately mixed with 5 mL of Free-SH buffer (containing 86 mmol/L Tris, 90 mmol/L glycine, 4 mmol/L EDTA, pH 8.0) and Total-SH buffer (containing 86 mmol/L Tris, 90 mmol/L glycine, 4 mmol/L EDTA, 8 mol/L urea, pH 8.0). 5 mL of the supernatant was collected after centrifuging at 8500 rpm for 15 min and was then combined with 40 μL of Ellman’s reagent (3 mg/mL DTNB in Tris-Gly buffer) by vigorous shaking. Absorbance was recorded at 412 nm after dark incubation for 30 min. The formula below was used to calculate the contents of -SH and S-S:

$$\text{-}\mathrm{SH}(\mu \mathrm{mol}/g)\;=\;\frac{75.35\;\times \;A\;\times \;D}{C}$$

$$S\text{-}S\left(\frac{\mu \mathrm{mol}}{g}\right)=\frac{{\text{-}\mathrm{SH}}_{\mathrm{Total}}-{\text{-}\mathrm{SH}}_{\mathrm{Free}}}{2}$$

where the A signifies the 412 nm absorbance reading, D is the dilution factor, C is the protein concentration, mg/mL.

### 2.9. Measurement of the Intermolecular Interactions

To determine the interactive forces in the formation and stability of SPI, PPI, SPBP, and SPCP samples, the method by Li, et al. [16] was applied. The solutions consisted of 0.6 mol/L NaCl, 6 mol/L urea, 0.5% SDS, and 0.05 mol/L DTT, prepared individually or in combination. Different solvents were used to mix 10 mg samples of SPI, PPI, SPBP, and SPCP, which were then allowed to stand for 1 h. Afterward, the samples were centrifuged at 8600 rpm for 25 min, and the protein concentration in the supernatant was measured using the biuret method.

### 2.10. Molecular Docking

The CDOCKER program in Discovery Studio 2019 was utilized for molecular docking analysis, adhering to the procedure outlined by Zhu, et al. [17]. The crystal structures of 7S in SPI (PDB: 3AUP), 11S in SPI (PDB: 1OD5), and legumin in PPI (PDB: 7U1I) were retrieved from the Protein Data Bank. The docking results were visualized using PyMOL 3.1, and 2D interaction diagrams were generated using LigPlot software (version 2.2, EMBL-EBI, Cambridge, UK).

### 2.11. Solubility Analysis

The solubility of SPI, PPI, SPBP, and SPCP was examined at pH 7.0 using the method provided by Tan, et al. [18]. Centrifugation of the sample (10 mg/mL) was performed at 9500 rpm for 18 min. The protein content in all sample dispersions was measured using the biuret method both before and after centrifugation. To perform the calculation, the following formula was utilized:

$$\mathrm{Solubility}\;(\%)\;=\;\frac{\mathrm{protein}\;\mathrm{content}\;\mathrm{of}\;\mathrm{the}\;\mathrm{supernatant}}{\mathrm{total}\;\mathrm{protein}\;\mathrm{content}\;\mathrm{before}\;\mathrm{centrifugation}}\;\times \;100\%$$

### 2.12. Rheological Measurements

The rheological properties of the SPI, PPI, SPBP and SPCP samples were assessed using a rotational rheometer (MARS40, Thermo Fisher Scientific Inc., MA, USA with parallel plates measuring 50 mm in diameter and a 1 mm gap. Samples of SPI, PPI, SPBP, and SPCP, each with equal volume, underwent temperature sweep tests on the rheometer plate. At a strain of 0.5% and an angular frequency of 10 Hz, the tests comprised three separate phases: starting with a 30 min heating phase from 25 to 95 °C, followed by a 10 min period maintained at 95 °C, and then a cooling phase from 95 to 25 °C over 12 min. The storage modulus (G′) and loss modulus (G″) were measured during the entire operation.

### 2.13. Statistical Analysis

Every test was conducted thrice, with the findings expressed as the mean ± standard deviation. Using SPSS 26.0, the data underwent ANOVA, with a significance level of *p* < 0.05. All the graphs were generated using Origin 2024 software.

## 3. Results and Discussion

### 3.1. SDS-PAGE Analysis

**Figure 1:**
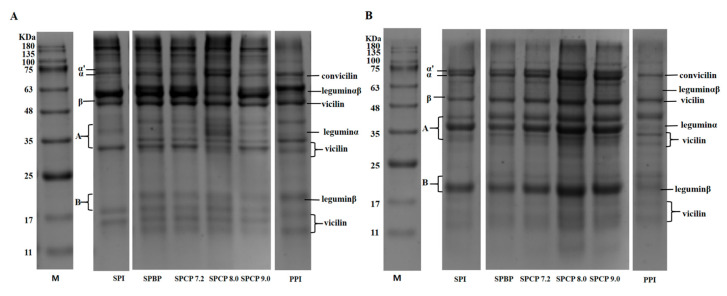
Non-reducing (**A**) and reducing (**B**) electrophoretic patterns of soy-pea blended protein and co-precipitated proteins.

The protein subunit compositions in SPI, PPI, SPBP and SPCP were analyzed via non-reducing and reducing SDS-PAGE. In general, the β-conglycinin subunits in SPI are primarily made up α (~68 kDa), α′ (~72 kDa), and β (~52 kDa), while the glycinin fraction includes an acidic subunit A (~35 kDa) and a basic subunit B (~20 kDa) [19]. The vicilin subunits in PPI are mainly composed α + β (~33 kDa) and α + β + γ (~50 kDa), and the legumin fraction consists of α (~40 kDa), β (~20 kDa), and α + β (~60 kDa) [20]. As shown in Figure 1A, under nonreducing conditions, both SPBP and SPCP exhibited typical bands of protein subunits that had been observed in SPI and PPI, suggesting that the major subunits from these proteins were present in the blended and co-precipitated proteins. Comparable findings were observed for tilapia-soybean protein co-precipitates [21]. Compared with SPI and PPI, protein bands of SPBP and SPCP were enhanced at the top of the electrophoretic, especially SPCP8.0, indicating that the presence of large molecular weight soluble protein in SPBP and SPCP. As shown in Figure 1B, Compared with SPBP, the intensities of the bands of α′, α, β, convicilin, vicilin, legumin α and legumin β subunits in SPCP were enhanced. This might result from stronger non-covalent caused by the co-precipitation of proteins from different sources [22]. Compared with the non-reducing electrophoresis, the bands at the top of SPBP and SPCP were weakened dramatically. This could be because adding DTT reduced the disulfide bonds between protein molecules, loosening their structure and causing the protein subunits to dissociate [23]. In summary, both SPBP and SPCP retained all the subunits present in the structure of SPI and PPI, while the co-precipitation treatment markedly strengthened the interactions of protein subunits.

### 3.2. FTIR Analysis

**Figure 2:**
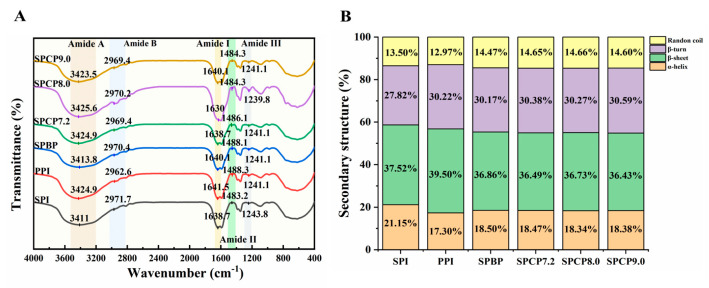
FTIR spectra (**A**), secondary structures (**B**) of soy-pea blended protein and soy-pea co-precipitated proteins.

Changes in the secondary structure of proteins can be observed using FTIR spectra. In Figure 2A, the amide A band in the range of 3200–3500 cm^−1^ was related to N-H stretching vibrations due to hydrogen bonding. The amide B band observed at 2970 cm^−1^ corresponded to C-H stretching. Meanwhile, the amide I and II bands associated with C=O stretching vibration, N-H bending, and C-N stretching were positioned at 1638 cm^−1^ and 1484 cm^−1^, respectively. At 1240 cm^−1^, the amide III band was linked to the bending of C-N and N-H [24]. The above characteristic peaks of SPBP and SPCP described were similar to those of SPI and PPI. However, the peak intensities of amide A, amide II, and amide III bands in SPCP8.0 were markedly stronger than those in the other proteins, and the amide B and amide I bands showed red-shifts. This might be due to the pH shift from alkaline to neutral, during which SPI and PPI underwent unfolding and refolding. This process allowed the hydrophobic groups originally buried within the molecule, to become more readily exposed and participate in interactions between the two proteins. Meanwhile, these structural alterations encouraged the creation of more intermolecular hydrogen bonds, which in turn strengthened both hydrogen bonding and hydrophobic interactions in the co-precipitated protein [25].

To further analyze the secondary structures of SPBP and SPCP, PeakFit v4.12 software was used for analysis in the amide I band in the spectra. This band consists of several components: 1645–1660 cm^−1^ (α-helix), 1610–1637 cm^−1^ and 1670–1690 cm^−1^ (β-sheet), 1660–1670 cm^−1^ (β-turn), and 1637–1645 cm^−1^ (random coil) [26]. In Figure 2B, SPI exhibited the high α-helix content (21.15%), while PPI contained a higher proportion of β-sheet structures (39.50%). The relative content of α-helix and β-sheet in SPBP and SPCP were lower than those in SPI, whereas the random coil content was higher than in both SPI and PPI. It is reported that α-helix and β-sheet contribute to protein structural order and compactness, whereas random coil confer conformational flexibility. The physical blending of SPI and PPI formed hydrogen bonds, which were relatively weak in strength, resulting in slight perturbations of the ordered structure of SPBP, a moderate increase in molecular flexibility, and limited transition toward more flexible and relaxed conformations. According to the study of Li, Chen, Hua, Yin, Zang, Yu and Zhang [8], the co-precipitation induced the unfolding of SPI and PPI through pH adjustment. Subsequently, the two proteins underwent synergistic folding and interacted closely, causing the peptide chains to rearrange and ultimately resulting in the formation of co-precipitated protein with specific secondary structures.

### 3.3. Intrinsic Fluorescence Spectroscopy

**Figure 3:**
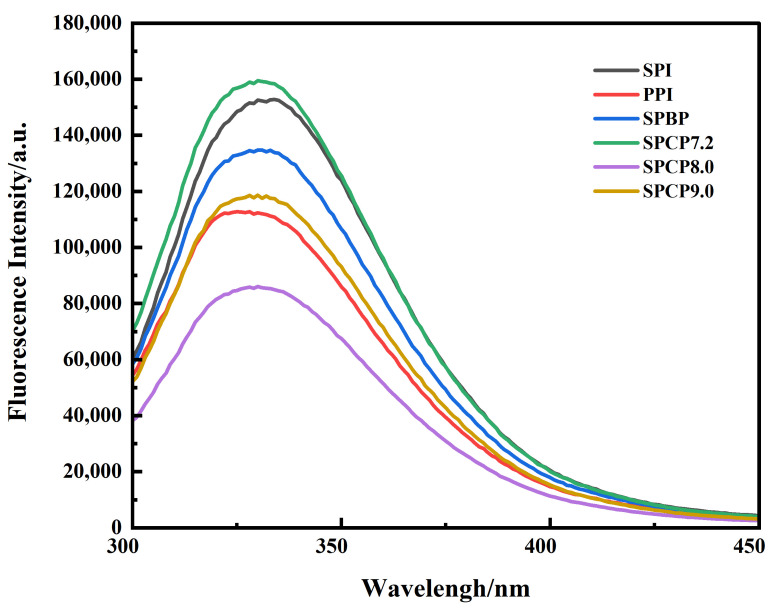
Fluorescence spectrogram of soy-pea blended protein and soy-pea co-precipitated proteins.

Intrinsic fluorescence spectroscopy is an effective indicator for probing tertiary structural changes in proteins [27]. In Figure 3, all samples exhibited varying fluorescence intensities at approximately 330 nm (the characteristic emission maximum of Trp residues). Among them, SPI exhibited a slight red-shift, PPI showed a slight blue-shift, while SPBP and SPCP showed no obvious variation. SPCP exhibited lower fluorescence intensity compared to SPBP (except for SPCP7.2). The observed difference might have resulted from stronger non-covalent interactions in the co-precipitated proteins. Wang, et al. [28] similarly observed decreased fluorescence intensity during the preparation of pine kernel protein–egg white protein co-precipitates. Meanwhile, the process of tuning the co-precipitated proteins back to neutrality from basicity, protein structure underwent rearrangements that not only reburied the tryptophan residues, but also changed their microenvironment, leading to a static fluorescence quenching [29]. In contrast, SPBP was merely a simple blend of SPI and PPI, in which the two proteins maintained their original conformations, leading to minimal changes in the microenvironment of Trp. Therefore, the fluorescence intensity of SPBP fell between those of SPI and PPI.

### 3.4. Particle Size and Zeta-Potential Analysis

**Figure 4:**
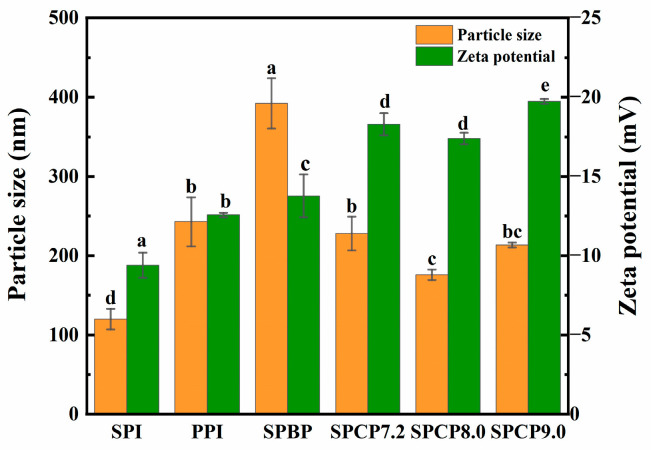
Particle size and zeta-potential of soy-pea blended protein and soy-pea co-precipitated proteins. Note: significant differences between samples are marked by different letters (*p* < 0.05).

In Figure 4, the average particle size of PPI (242.9 nm) was larger than that of SPI (120 nm). SPBP (392.2 nm) exhibited the largest average particle size. In contrast, the average particle sizes of SPCP7.2 (228 nm), SPCP8.0 (176.1 nm) and SPCP9.0 (213.5 nm) were significantly smaller than that of SPBP. Some studies had demonstrated that protein–protein molecule interactions could reduce the particle size of protein samples [30]. During the co-precipitation process, interactions between SPI and PPI promoted closer packing of protein molecules, thus reducing the particle size of SPCP. In addition, SPBP showed weak intermolecular interactions and a looser structure, leading to a larger average particle size. The results of zeta-potential further supported this conclusion. The absolute values of the zeta-potential of SPCP7.2 (−18.3 mV), SPCP8.0 (−17.4 mV) and SPCP9.0 (−19.7 mV) notably greater than those for SPI, PPI and SPBP (−13.7 mV) samples. This demonstrated that co-precipitation increased the surface charge density of protein molecules and enhanced electrostatic repulsion, thereby promoting orderly molecular packing through optimized protein–protein interactions and consequently maintaining smaller and more uniform particle sizes [31]. These characteristics make SPCP particularly advantageous for food applications requiring uniform texture and optimized functionality, such as plant-based meat products.

### 3.5. H_0_ Analysis

**Figure 5:**
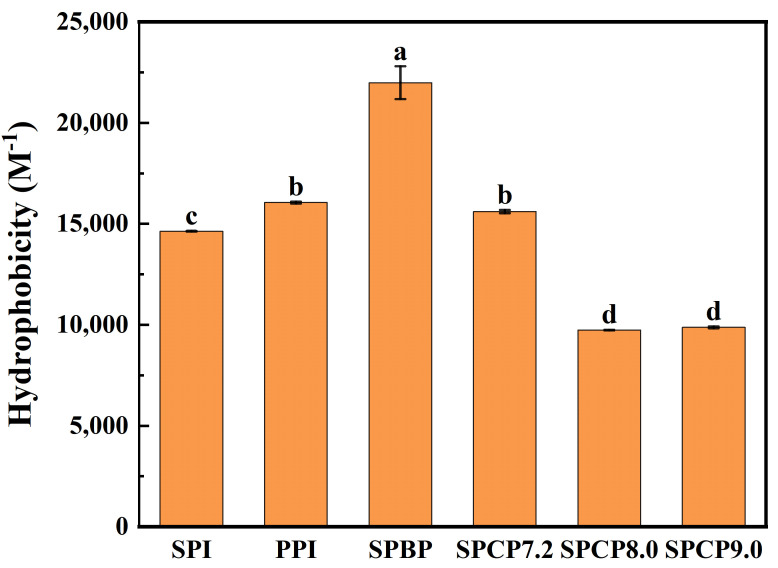
Surface hydrophobicity of soy-pea blended protein and soy-pea co-precipitated proteins. Note: significant differences between samples are marked by different letters (*p* < 0.05).

Hydrophobic amino acids on the side chains of a protein determine its H_0_, and an appropriate H_0_ is crucial for the protein’s solubility and functional characteristics (such as gel-forming ability) [32]. As shown in Figure 5, the H_0_ of PPI was greater than that of SPI, SPBP (21,987.3) exhibited the highest H_0_, whereas SPCP7.2 (15,607.3), SPCP8.0 (9744.8) and SPCP9.0 (9875.5) displayed significantly reduced H_0_ compared with SPBP. This may be attributed to hydrophobic interaction-driven co-assembly between SPI and PPI during co-precipitation process, which led to the burying of previously exposed hydrophobic residues inside the proteins again, thereby forming a more stable molecular structure. In addition, the phenomenon in which interactions between heterologous proteins bury hydrophobic groups was known as “hydrophobic collapse” [33], which was particularly prominent in SPCP8.0 and SPC9.0. In contrast, SPBP exhibited the highest H_0_, which might have originated from the SPBP being simply a mixture of SPI and PPI, where the constituent proteins retained their native conformations. It not only preserved the relatively large number of hydrophobic amino acids (such as phenylalanine and leucine) on the surface of PPI but also prevented a decrease in the exposure of hydrophobic groups due to interactions, resulting in its highest H_0_ [34].

### 3.6. -SH and S-S Analysis

**Figure 6:**
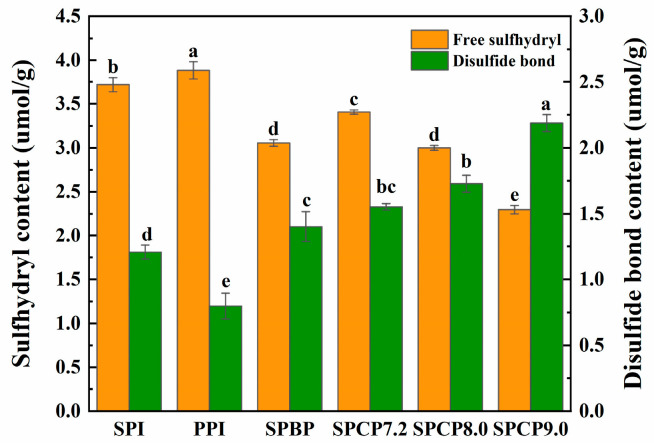
The content of -SH and S-S in soy-pea blended and co-precipitated proteins. Note: significant differences between samples are marked by different letters (*p* < 0.05).

Under specific conditions, -SH and S-S can transform into each other, and play a crucial role in maintaining the tertiary structure of proteins [35]. Figure 6 showed that, compared with SPI and PPI, both SPBP and SPCP exhibited decreased free -SH content and increased S-S content. In SPBP, the observed increase in S-S may primarily originate from the intrinsic S-S structures of 11S globulin in SPI and legumin in PPI. In contrast, SPCP underwent alkaline pH treatment that induced protein unfolding into a molten globule state and exposed previously buried -SH groups. These exposed -SH groups facilitated the interchange reaction of -SH to form S-S under alkaline conditions, thereby resulting in a decrease in -SH content [36]. In SPCP9.0, the stronger alkaline environment caused more extensive protein unfolding, exposing more internal -SH groups and subsequently forming S-S bonds. While the structural stability of the protein was enhanced, excessive S-S cross-linking might have resulted in an overly rigid network structure, ultimately compromising the structural uniformity and functional properties (e.g., solubility and gel elasticity) of the protein [37]. In contrast, SPCP8.0 likely formed a moderate S-S cross-linking. This balanced intermolecular force not only enhanced protein structural stability but also maintained sufficient structural plasticity.

### 3.7. Molecular Interaction of Proteins

**Figure 7:**
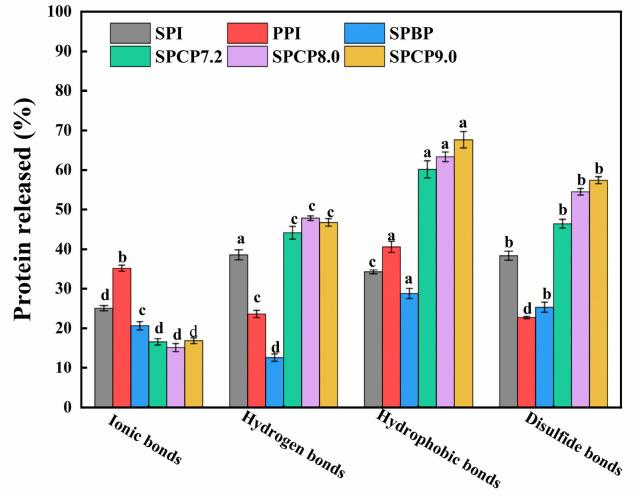
Intermolecular interactions of soy-pea blended protein and soy-pea co-precipitated proteins. Note: significant differences between samples are marked by different letters (*p* < 0.05).

In order to shed light on the interaction force between SPI and PPI, this study examined the types of protein–protein interactions. Specifically, ionic bonds, hydrogen bonds, hydrophobic interactions, and disulfide bonds were disrupted by NaCl, urea, SDS, and DTT, respectively [38]. As shown in Figure 7, hydrophobic interactions, hydrogen bonds, and S-S played dominant roles in stabilizing the SPCP structure. In contrast, SPBP exhibited significantly weaker intermolecular interactions. Compared with SPI, PPI, and SPBP, SPCP showed significantly enhanced hydrophobic interactions, which might be attributed to the optimal spatial matching of hydrophobic regions between SPI and PPI during co-precipitation. This matching maintained the intramolecular hydrophobic core and promoted intermolecular hydrophobic packing, synergistically enhancing hydrophobic interactions. Additionally, the increased proportion of hydrogen bonds in SPCP indicated that it not only retained the native hydrogen bond network of SPI but also formed new intermolecular hydrogen bonds with PPI. Meanwhile, the formation of S-S further stabilized the SPCP structure, which was consistent with the -SH and S-S analysis results, confirming the conversion of partial -SH into S-S during co-precipitation. However, ionic bonds were weaker in SPCP, likely due to the overall negative charge of the system under pH regulation (in Figure 4), resulting in electrostatic repulsion that inhibited the formation of ionic bonds. By contrast, SPBP exhibited weaker intermolecular interactions. This phenomenon might be attributed to the electrostatic repulsion between negative charges of SPI and PPI. This repulsion prevented close intermolecular contact, limiting the exposure of functional groups (e.g., hydrophobic residues and -SH) and exert their activity. Consequently, only weak interactions between SPI and PPI (e.g., localized electrostatic interactions and van der Waals forces) were formed rather than stronger hydrophobic interactions, hydrogen bonds, or S-S bonds. This also validated the unique efficacy of co-precipitation technology in synergistically enhancing the structural complementary effects of SPI and PPI through multiple intermolecular forces.

### 3.8. Molecular Docking Analysis

SPI is primarily composed of 7S and 11S globulins, accounting for approximately 70% of the SPI [39]. In this study, 7S and 11S globulins were selected as receptor proteins. PPI mainly consisted of vicilin and legumin, wherein vicilin in PPI existed as a trimer that lacked cysteine residues and contained more peptide segments, making it unsuitable as a model [40]. In contrast, legumin, with its hexameric structure, contributed more significantly to the binding capacity of PPI and was therefore chosen as the docking receptor. After obtaining protein structures from the PDB database, water molecules, hydrogen atoms, and non-target proteins were removed. Molecular docking experiments were then performed using CDOCKER to further explore the potential interaction mechanisms between SPI and PPI.

**Figure 8:**
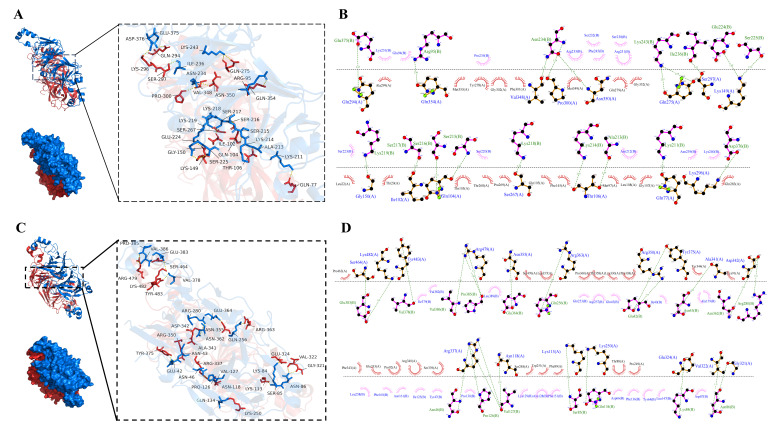
The binding mode of 7S of SPI and legumin of PPI (**A**) and the Ligplot diagram of the interaction between 7S and legumin (**B**); The binding mode of 11S of SPI and legumin of PPI (**C**) and the Ligplot diagram of the interaction between 11S and legumin (**D**). The green dashed lines represent hydrogen bonds, while arc-shaped eyelash patterns indicate hydrophobic interactions.

Figure 8A displayed the optimal docking conformation of 7S-legumin (binding energy: −14.5 kcal/mol), where the red 7S protein and blue legumin protein formed extensive contacts. The 2D schematic diagram (Figure 8B) revealed interactions where, on one hand, hydrophobic amino acids such as Ala299, Leu22, Phe245, Tyr278, and Met353 in 7S, and Ser232, Lys251, Arg233, Pro235, and Asn239 in legumin formed extensive hydrophobic interactions that maintained the 3D structure of the complex. On the other hand, hydrogen bonds were formed between Val348, Pro300, and Asn350 of 7S and Asn234 of legumin; Lys149 and Glu224, Ser225; Gln104 and Ser215, Ser216; Thr106 and Lys214, Ala213; as well as between Gln275, Ser297, Gly150, Ile102 and Lys243, Ile236, Lys219, Ser217, respectively. Ranging from 2.62 to 3.30 Å, these hydrogen bonds reinforced non-covalent bond strength and stabilized the complex’s spatial conformation.

Figure 8C showed the optimal docking conformation of 11S-legumin (binding energy: −16.1 kcal/mol), exhibiting an interaction pattern similar to that of 7S-legumin. As illustrated in Figure 8D, hydrophobic interactions were formed between Thr358, Lys330, Pro338, Tyr344, Lys91 of 11S and Ile379, Val382, Leu389, Gln253, Asp257 of legumin. Additionally, a more extensive hydrogen bond network was established, including interactions between Ser464, Lys482 of 11S and Glu383 of legumin; Arg479 and Val386, Pro385; Arg350, Tyr375 and Glu42; Asp342 and Asn362, Arg280; Arg337, Asn118, and Asn46, Pro126, Val127; Val322, Gly321, and Asn86, as well as between Tyr483, Asn355, Arg363, Ala341 and Val378, Glu364, Gln256, Asn43, respectively, with bond lengths ranging from 2.59 to 3.81 Å. These results were consistent with FTIR analysis, confirming that SPI and PPI formed stable complexes through hydrophobic interactions and hydrogen bonds. The hydrogen bond distances mostly within 2.6–3.8 Å indicate strong binding strength [41]. Among them, the 11S-legumin complex (−16.1 kcal/mol) demonstrated greater binding affinity than the 7S-legumin complex (−14.5 kcal/mol).

### 3.9. Solubility

**Figure 9:**
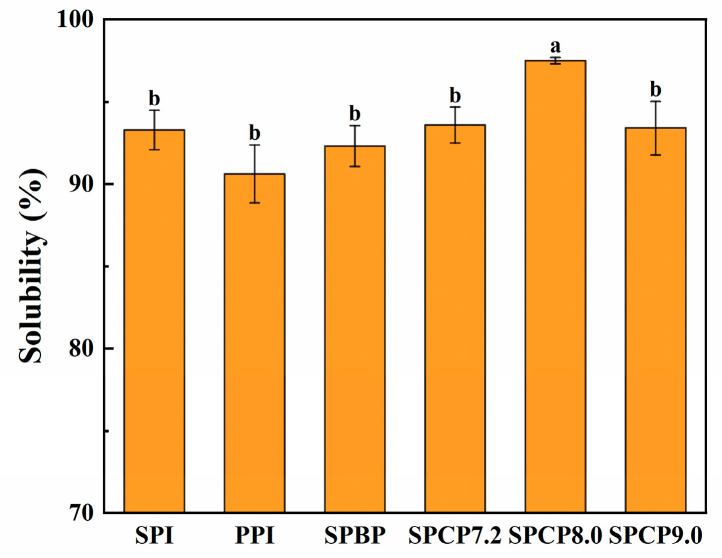
Solubility of soy-pea blended protein and soy-pea co-precipitated proteins. Note: significant differences between samples are marked by different letters (*p* < 0.05).

The solubility of proteins is essential for determining their functional properties [42]. As shown in Figure 9, the solubility of SPI (93.26%) was relatively higher than that of PPI (90.60%), and the solubility of SPCP7.0, SPCP8.0 and SPCP9.0 was 93.56%, 97.50%, and 93.39%, respectively, which was slightly higher than that of SPBP (92.29%). Among them, SPCP8.0 exhibited the highest solubility. At alkaline environment, SPI and PPI formed a special intermediate state called the molten globule phenomenon due to the solution’s ionic strength. In this state, reduced side-chain interactions increased flexibility and enhanced protein–water binding, thereby improving its solubility [43]. When pH was adjusted back to neutral, the proteins underwent hydrophobic collapse, re-burying hydrophobic residues and redistributing surface polar groups to form a stable structure with high charge density [44], as evidenced by the significantly increased zeta-potential absolute values in Figure 4, indicating enhanced intermolecular electrostatic interactions. This strengthened hydration (ion-dipole interactions) and further improved solubility. These findings were in agreement with Zhang, et al. [45], who reported higher solubility for soy-whey co-precipitated proteins than for single proteins. However, SPCP9.0 might have exhibited increased particle size, which resulted in partial burial of polar groups within the interior, consequently reducing their interactions with water molecules. In contrast to SPCP, SPBP preserved the native structures of SPI and PPI, in which the proteins were merely associated through weak intermolecular interactions. Thus, it ultimately resulted in inferior solubility enhancement effect being slightly lower than that of SPCP.

### 3.10. Rheological Analysis

**Figure 10:**
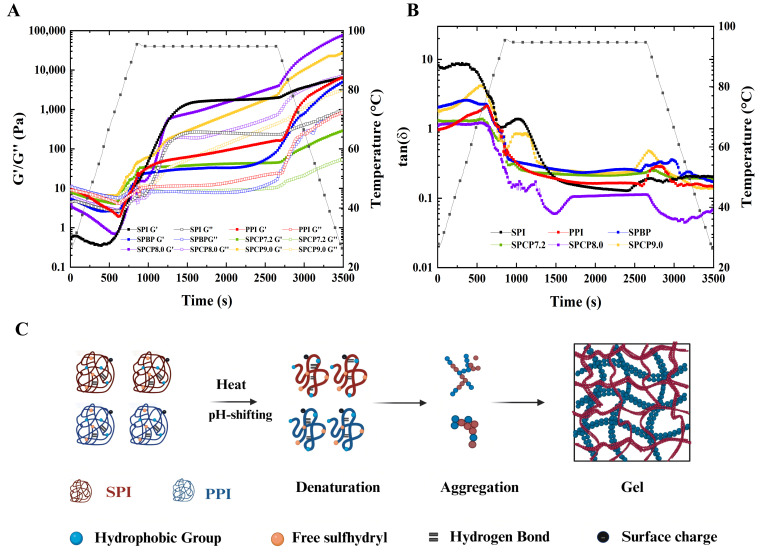
The storage modulus (G′), loss modulus (G″) (**A**) and loss tangents (tan δ) (**B**) of soy-pea blended protein and soy-pea co-precipitated proteins, and a schematic representation of gelation (**C**) of soy-pea co-precipitated proteins.

Rheology is an extremely important indicator of protein gel properties. Figure 10A showed the temperature sweeping patterns of SPI, PPI, SPBP, and SPCP during thermal gelation, and Figure 10C illustrated the schematic diagram of the gel formation process of SPCP. Here, the storage modulus (G′) reflected the elastic property of the gel network, and the loss modulus (G″) represented its viscoelasticity. In the early heating phase, G′ was consistently lower than G″. When the temperature reached approximately 90 °C, G′ surpassed G″, indicating the onset of protein denaturation, followed by aggregation and a transition from sol to gel. With a further temperature rise, the G′ of SPI increased significantly and became higher than that of PPI. For SPCP, the unfolding of SPI and PPI exposed -SH and hydrophobic groups, leading to rapid irreversible aggregation via S-S and hydrophobic interactions, thereby forming a preliminary gel network. Continued heating induced further structural rearrangement of the gel network, possibly involving the fusion of protein aggregates within the chains, which enhanced intermolecular interactions and formed a denser three-dimensional network structure, manifesting as a notable increase in G′. In the holding stage, except for SPCP7.2, the G′ of the SPCP was between that of SPI and PPI, which reflected that the gelation is in a transitional state. During the cooling phase (95 °C to 25 °C over 12 min), G′ and G″ for all samples continued to increase (G′ > G″). The G′ of SPCP (except for SPCP7.2) exhibited a more pronounced increase, which might be attributed to the reduction of system entropy that promoted protein intermolecular interactions, consequently forming a more stable elastic gel network [46]. However, SPCP7.2 showed poorer G′, likely due to insufficient protein unfolding at pH 7.2. SPCP8.0 had the highest G′, indicating that SPCP8.0 had a more outstanding gel-forming ability and excellent application potential in plant-based meat products. Its robust and elastic gel network is crucial for accurately mimicking the texture and mouthfeel of real meat. In contrast, SPBP, due to the inherent structural differences between SPI (rigid structure) and PPI (loose structure), failed to promote protein conformational changes via physical blending, resulting in weaker intermolecular interactions (such as localized hydrophobic contacts and hydrogen bonds). This yielded a relatively loose and uneven gel network structure (the G′ was always lower than that of all SPCP samples except SPCP7.2) and relatively poor gel performance.

The loss tangent (tan δ = G″/G′) reflected the relative ratio of viscoelasticity and its dynamic changes during gel formation. When the G′ was greater than the G″, it indicated gel behavior of the protein sample, and thus tan δ = 1 (gel point) was generally considered the onset of gelation [47]. As shown in Figure 10B, the tan δ of all samples further decreased (indicating enhanced elasticity), and the final tan δ were all below 1, suggesting that their gels exhibited more elastic rather than viscous behavior. Among them, SPCP8.0 showed the lowest tan δ value and was the first to drop below 1, indicating the formation of a denser elastic network structure. This might be attributed to the synergistic optimization of hydrophobic interactions, hydrogen bonds, and S-S bonds under pH regulation, which endowed it with rapid gelation capability and excellent rheological properties. In food systems (such as plant-based meat products), this characteristic could not only optimize the processing technology by reducing heat input or shortening processing time but also mimic the texture and mouthfeel of animal meat, providing new ideas for the development of high-quality plant protein products.

## 4. Conclusions

This study demonstrated that the physically blended SPBP failed to induce synergistic conformational changes between SPI and PPI (such as hydrophobic core rearrangement and S-S exchange), only forming localized molecular contacts (e.g., transient hydrogen bonds and van der Waals forces), which hindered structural optimization. In contrast, the SPCP prepared by co-precipitation (particularly SPCP8.0) underwent structural adjustment, with modified surface properties. Experimental and docking analyses confirmed the stability of SPCP relied mainly on hydrophobic interactions, hydrogen bonds, and S-S bonds. The synergistic effects of these forces not only enhanced solubility (with SPCP8.0 reaching 97.50%), but also led to SPCP8.0 exhibiting the highest storage modulus (G′), loss modulus (G″) and the lowest tan δ, indicating the formation of a denser and more elastic three-dimensional network structure. The superior performance of SPCP8.0 also suggested that moderate alkaline conditions during co-precipitation balanced protein unfolding/refolding, yielding optimal structural and functional characteristics. Consequently, the pH-driven co-precipitation technique effectively utilized the structural complementarity between SPI and PPI by inducing molecular reorganization and enhancing interactions. These findings not only established a theoretical foundation for optimizing other plant-based dual-protein combinations but also provided an ideal scientific basis for the development of high-performance plant-based meat products (particularly those capable of mimicking the texture, chewiness, and resilience of real meat).

## Data Availability

The original contributions presented in this study are included in the article. Further inquiries can be directed to the corresponding author.

## References

[1] Lee J.-S., Kim S., Jeong Y.J., Choi I., Han J. (2023). Impact of interactions between soy and pea proteins on quality characteristics of high-moisture meat analogues prepared via extrusion cooking process. Food Hydrocoll..

[2] Lei Y., Yue J., Min T., Cheng C., Weng S., Long Y., Luo Y., Zheng Y., Wan Y. (2025). The strengthening effects of different types of salt on the mechanical properties of soy protein isolate and pea protein isolate composite gels. Food Hydrocoll..

[3] Kristensen H.T., Denon Q., Tavernier I., Gregersen S.B., Hammershøj M., Van der Meeren P., Dewettinck K., Dalsgaard T.K. (2021). Improved food functional properties of pea protein isolate in blends and co-precipitates with whey protein isolate. Food Hydrocoll..

[4] Youssef A.M., Abu-Foul N.S., Moharram Y.G. (1995). Preparation and characteristics of coprecipitate proteins from oilseeds and legumes seeds. Food Nahr..

[5] Shao F., Zhang Y., Wan X., Duan Y., Cai M., Hu K., Zhang H. (2024). Molecular regulation of rapeseed protein for improving its techno-functional properties. Int. J. Biol. Macromol..

[6] Tian T., Tong X., Ren K., Cao J., Yuan Y., Yang J., Zhu J., Miao L., Yang S., Yu A. (2022). Influence of protein ratios on the structure and gel properties of soybean-wheat co-precipitated proteins. LWT.

[7] Zhou X., Zhang C., Zhao L., Cao W., Zhou C., Xie X., Chen Y. (2022). Functionality of Pea-Grass Carp Co-Precipitated Dual-Protein as Affected by Extraction pH. Foods.

[8] Li J., Chen Y., Hua X., Yin L., Zang J., Yu W., Zhang T. (2024). Soy protein isolate with higher solubility and improved gel properties when co-assembled with walnut protein. Int. J. Food Sci. Technol..

[9] Choi H.W., Choi M., Ryoo C., Hahn J., Choi Y.J. (2024). Viscometry-based prediction of structural properties of high-moisture meat analogues using gelation properties of soy and pea isolate protein blends. Food Hydrocoll..

[10] Wang N., Zhou X., Wang W., Wang L., Jiang L., Liu T., Yu D. (2021). Effect of high intensity ultrasound on the structure and solubility of soy protein isolate-pectin complex. Ultrason. Sonochem..

[11] Zhao Y.-R., Peng N., Wang C., Li Y.-Q., Liang Y., Guo Z.-W., Sun A.-Y., Ren X. (2024). Preparation and characterization of pea protein isolate-egg white protein composite gels. Food Hydrocoll..

[12] Ma X., Hou F., Zhao H., Wang D., Chen W., Miao S., Liu D. (2020). Conjugation of soy protein isolate (SPI) with pectin by ultrasound treatment. Food Hydrocoll..

[13] Dong D., Cui B. (2021). Fabrication, characterization and emulsifying properties of potato starch/soy protein complexes in acidic conditions. Food Hydrocoll..

[14] Wang R., Wang T., Feng W., Wang Q., Wang T. (2021). Rice proteins and cod proteins forming shared microstructures with enhanced functional and nutritional properties. Food Chem..

[15] Kang Z.-L., Bai R., Lu F., Zhang T., Gao Z.-S., Zhao S.-M., Zhu M.-M., Ma H.-J. (2022). Effects of high pressure homogenization on the solubility, foaming, and gel properties of soy 11S globulin. Food Hydrocoll..

[16] Li Y., Wu X., Wu Z., Kong Y., Kang Z., Xie F., Sun L. (2024). Formation of thermal-induced microgels from soy protein hydrolysates: Effects of selective proteolysis on glycinin/β-conglycinin. Int. J. Biol. Macromol..

[17] Zhu P.-y., Ma C.-m., Yang Y., Bian X., Ren L.-k., Wang B., Liu X.-f., Chen F.-l., Zhang G., Zhang N. (2024). Elucidating the interaction mechanism of rice glutelin and soybean 11S globulin using multi-spectroscopy and molecular dynamics simulation methods. Food Chem..

[18] Tan M., Xu J., Gao H., Yu Z., Liang J., Mu D., Li X., Zhong X., Luo S., Zhao Y. (2021). Effects of combined high hydrostatic pressure and pH-shifting pretreatment on the structure and emulsifying properties of soy protein isolates. J. Food Eng..

[19] Li Q., Shen F., He X., Xing C., Yan W., Fang Y., Hu Q. (2023). Modification of soy protein isolate using dielectric barrier discharge cold plasma assisted by modified atmosphere packaging. Food Chem..

[20] Tang Q., Roos Y.H., Miao S. (2023). Plant Protein versus Dairy Proteins: A pH-Dependency Investigation on Their Structure and Functional Properties. Foods.

[21] Liu Q., Tan L., Hong P., Liu H., Zhou C. (2024). Tilapia-soybean protein co-precipitates: Focus on physicochemical properties, nutritional quality, and proteomics profile. Food Chem. X.

[22] Kristensen H.T., Christensen M., Hansen M.S., Hammershøj M., Dalsgaard T.K. (2021). Protein–protein interactions of a whey–pea protein co-precipitate. Int. J. Food Sci. Technol..

[23] Wang Y., Chen X., Xu X., Du M., Zhu B., Wu C. (2022). Disulfide bond-breaking induced structural unfolding and assembly of soy protein acting as a nanovehicle for curcumin. Innov. Food Sci. Emerg. Technol..

[24] Shrestha S., van ‘t Hag L., Haritos V., Dhital S. (2023). Comparative study on molecular and higher-order structures of legume seed protein isolates: Lentil, mungbean and yellow pea. Food Chem..

[25] Wang X., Chen C., Bao Y., Wang Y., Leonidovna Strakh Y. (2024). Encapsulation of three different types of polyphenols in casein using a customized pH-driven method: Preparation and characterization. Food Res. Int..

[26] Zhao X., Chen F., Xue W., Lee L. (2008). FTIR spectra studies on the secondary structures of 7S and 11S globulins from soybean proteins using AOT reverse micellar extraction. Food Hydrocoll..

[27] Vivian J.T., Callis P.R. (2001). Mechanisms of Tryptophan Fluorescence Shifts in Proteins. Biophys. J..

[28] Wang J., Huang W., Wang X., Zhao Y., Zhang L. (2025). Modulating the gelatinization and retrogradation characteristics of corn starch by controlling pine kernel protein/egg white protein thermal co-aggregation. Carbohydr. Polym..

[29] Cahyana Y., Gordon M.H. (2013). Interaction of anthocyanins with human serum albumin: Influence of pH and chemical structure on binding. Food Chem..

[30] Sun Y., Wang L., Wang H., Zhou B., Jiang L., Zhu X. (2025). Effect of pH-shifting and ultrasound on soy/potato protein structure and gelation. Food Hydrocoll..

[31] Jiang S., Ding J., Andrade J., Rababah T.M., Almajwal A., Abulmeaty M.M., Feng H. (2017). Modifying the physicochemical properties of pea protein by pH-shifting and ultrasound combined treatments. Ultrason. Sonochem..

[32] Nakai S. (1983). Structure-function relationships of food proteins: With an emphasis on the importance of protein hydrophobicity. J. Agric. Food Chem..

[33] Chi E.Y., Krishnan S., Randolph T.W., Carpenter J.F. (2003). Physical Stability of Proteins in Aqueous Solution: Mechanism and Driving Forces in Nonnative Protein Aggregation. Pharm. Res..

[34] Guo X., Wu X., Sun Z., Li D., Jia H., Zhang K., Zhao Y., Zheng H. (2025). Preparation, characterization, and binding mechanism of pH-driven gliadin/soy protein isolate nanoparticles. Food Res. Int..

[35] Kang Z., Zhang S., Kong Y., Wu Z., Li Y., Liu T., Xie F. (2024). Modification of soybean protein isolate by pH-shifting combined with ultrasonic treatment: Structural, viscosity, and functional properties. Food Struct..

[36] Sun Y., Chen H., Chen W., Zhong Q., Shen Y., Zhang M. (2022). Effect of ultrasound on pH-shift to improve thermal stability of coconut milk by modifying physicochemical properties of coconut milk protein. LWT.

[37] Cheng J., Li Z., Wang J., Zhu Z., Yi J., Chen B., Cui L. (2022). Structural characteristics of pea protein isolate (PPI) modified by high-pressure homogenization and its relation to the packaging properties of PPI edible film. Food Chem..

[38] Yang Y., Wang Q., Lei L., Li F., Zhao J., Zhang Y., Li L., Wang Q., Ming J. (2020). Molecular interaction of soybean glycinin and β-conglycinin with (−)-epigallocatechin gallate induced by pH changes. Food Hydrocoll..

[39] Zheng L., Regenstein J.M., Zhou L., Wang Z. (2022). Soy protein isolates: A review of their composition, aggregation, and gelation. Compr. Rev. Food Sci. Food Saf..

[40] Cheng T., Sun Z., Zheng X., Zhang J., Hu Z., Liu R., Guo Z., Wang Z. (2025). Explore the binding mechanism and dynamic variation of pea protein isolate-rutin complexes to effectively improve the foam performance of pea protein isolate. Food Chem..

[41] Xu L., Wang T., Shan Y., Wang R., Yi C. (2024). Soybean protein isolate inhibiting the retrogradation of fresh rice noodles: Combined experimental analysis and molecular dynamics simulation. Food Hydrocoll..

[42] Du M., Xie J., Gong B., Xu X., Tang W., Li X., Li C., Xie M. (2018). Extraction, physicochemical characteristics and functional properties of Mung bean protein. Food Hydrocoll..

[43] Jiang J., Chen J., Xiong Y.L. (2009). Structural and Emulsifying Properties of Soy Protein Isolate Subjected to Acid and Alkaline pH-Shifting Processes. J. Agric. Food Chem..

[44] Chen M., Lu J., Liu F., Nsor-Atindana J., Xu F., Goff H.D., Ma J., Zhong F. (2019). Study on the emulsifying stability and interfacial adsorption of pea proteins. Food Hydrocoll..

[45] Zhang X., Qi B., Xie F., Hu M., Sun Y., Han L., Li L., Zhang S., Li Y. (2021). Emulsion stability and dilatational rheological properties of soy/whey protein isolate complexes at the oil-water interface: Influence of pH. Food Hydrocoll..

[46] Lv Y., Xu L., Tang T., Li J., Gu L., Chang C., Zhang M., Yang Y., Su Y. (2023). Gel properties of soy protein isolate-potato protein-egg white composite gel: Study on rheological properties, microstructure, and digestibility. Food Hydrocoll..

[47] Li X., Chen L., Hua Y., Chen Y., Kong X., Zhang C. (2020). Effect of preheating-induced denaturation during protein production on the structure and gelling properties of soybean proteins. Food Hydrocoll..

